# Impairment of bimanual in-phase movement during recovery from frontal lobe tumor surgery: a case report

**DOI:** 10.3389/fnins.2023.1217430

**Published:** 2023-09-28

**Authors:** Kozue Takada, Takuya Yamaguchi, Yuko Hyuga, Yuto Mitsuno, Satoshi Horiguchi, Masako Kinoshita, Takeshi Satow

**Affiliations:** ^1^Department of Neurology, National Hospital Organization Utano National Hospital, Kyoto, Japan; ^2^Department of Rehabilitation, Nagahama City Hospital, Nagahama, Shiga, Japan; ^3^Department of Neurosurgery, Nagahama City Hospital, Nagahama, Shiga, Japan; ^4^Department of Neurosurgery, National Hospital Organization Utano National Hospital, Kyoto, Japan

**Keywords:** bimanual movement, frontal lobe, primary motor cortex, supplementary motor area, premotor cortex, motor coordination

## Abstract

The mechanisms underlying bimanual coordination have not yet been fully elucidated. Here, we evaluated the clinical features of bimanual movement impairment in a patient following surgery for a frontal lobe tumor. The patient was an 80-year-old man who had undergone subtotal tumor resection for a tumor in the right superior frontal gyrus. Histological examination of the resected specimen led to the diagnosis of malignant lymphoma of the diffuse large B-cell type, and the patient subsequently received high-dose methotrexate-based chemotherapy. Postoperatively, the patient had difficulty with bimanual movement, and on the 5th postoperative day we found that the impairment could not be attributed to weakness. Temporal changes in the characteristics of manual movements were analyzed. Bimanual diadochokinesis (opening/closing of the hands, pronation/supination of the forearms, and sequential finger movements) was more disturbed than unilateral movements; in-phase movements were more severely impaired than anti-phase movements. Bimanual movement performance was better when cued using an auditory metronome. On the 15th postoperative day, movements improved. The present observations show that in addition to the disturbance of anti-phase bimanual movements, resection of the frontal lobe involving the supplementary motor area (SMA) and premotor cortex (PMC) can cause transient impairment of in-phase bimanual diadochokinesis, which can be more severe than the impairment of anti-phase movements. The effect of auditory cueing on bimanual skills may be useful in the diagnosis of anatomical localization of the superior frontal gyrus and functional localization of the SMA and PMC and in rehabilitation of patients with brain tumors, as in the case of degenerative movement disorders.

## Graphical Abstract

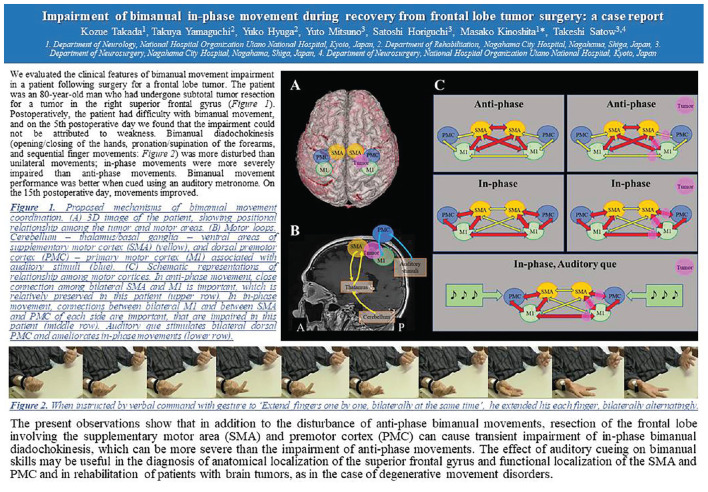

## 1. Introduction

Bimanual coordination remains unclear in terms of its underlying mechanisms. In addition to the primary motor cortex, the supplementary motor area (SMA) and premotor cortex (PMC) play significant roles in complex motor control. The SMA and PMC reside in the posterior part of the superior frontal gyrus and in the mesial and lateral portions of Brodmann area 6, respectively. SMA syndrome is characterized by akinesia with preserved muscle strength, which is more severe on the contralateral side of the SMA lesion and usually recovers over several weeks (Laplane et al., 1977). The disturbance of anti-phase bimanual alternating movements is a residual symptom of SMA syndrome (Laplane et al., 1977).

Here, we report the case of a patient with a frontal lobe tumor who showed a rare manifestation of more severe impairment of in-phase than of anti-phase movements after surgery.

## 2. Case description

**FIGURE 1:**
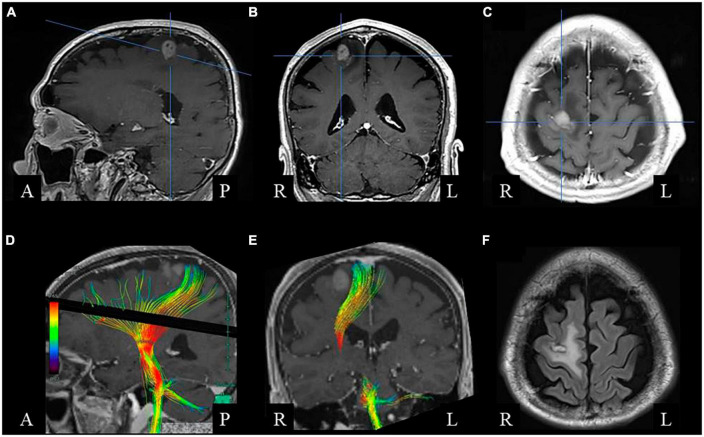
Brain magnetic resonance imaging before surgery. Gadolinium-enhanced T1-weighted sagittal **(A)**, coronal **(B)**, and axial **(C)** images show a right frontal lesion with enhancement. **(D,E)** Diffusion tensor image showing the corticospinal tract. **(F)** Postoperative fluid-attenuated inversion recovery axial image, showing small resected area with low-intensity rim surrounded by high-intensity white matter lesion.

An 80-year-old right-handed man underwent subtotal resection of a tumor in the right superior frontal gyrus (Figures 1A-C). The tumor was histologically diagnosed as a diffuse large B-cell type malignant lymphoma. and he subsequently received high-dose, methotrexate-based chemotherapy. The tumor did not involve the pre- and postcentral gyri. Diffusion tensor imaging showed that corticospinal tract was intact (Figures 1D, E). Thus, the primary sensorimotor cortex was preserved (Figure 1F). Postoperatively, the patient had difficulty with bimanual movement. At first, we considered that the difficulty was caused by weakness and akinesia. However, on the 5th postoperative day we found that the impairment could not be attributed to weakness because his motor skills varied depending upon the type of movement. Thus, we precisely evaluated his movement in the recovery process. This study was conducted according to the principles of the Declaration of Helsinki. The Institutional Ethics Review Board waived approval of the study design. Written informed consent was obtained from the patient for publication of the case report and accompanying images.

## 3. Diagnostic assessment

Temporal changes in the characteristics and performance of manual movements were analyzed by reviewing video recordings (Supplementary Videos 1–13).

**FIGURE 2:**
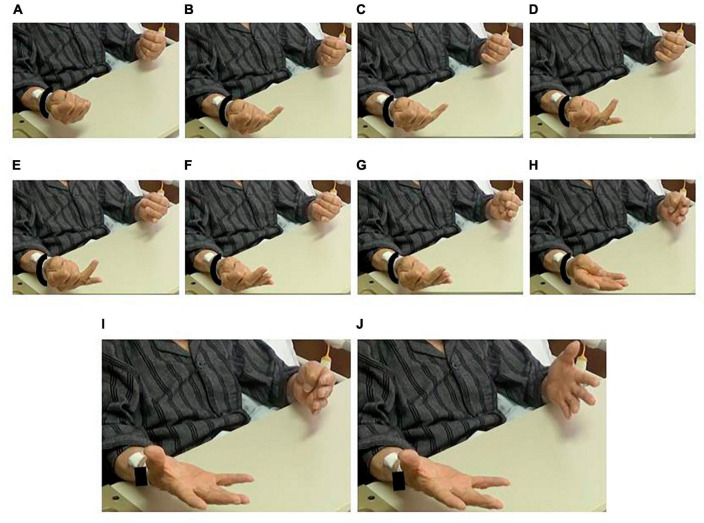
Impairment of in-phase movement. Despite an instruction for simultaneous movement, the patient extended his fingers alternatingly. **(A)** Fists. Right-to-left extension of panels **(B,C)** small fingers, **(D,E)** ring fingers, **(F,G)** tall fingers, **(H)** index fingers, and **(I,J)** thumbs. See Supplementary Video 6.

On the 5th postoperative day, the unilateral left-sided movements were skillful (Supplementary Videos 1, 3, 5). In contrast, when the patient was instructed to perform bilateral movements of the hands and arms, such as raising the arms, pronation and supination of the forearms, and opening and closing of the hands, he could not move his left side properly (Supplementary Videos 2, 4, and 6). When an instruction on in-phase sequential finger movement was given by verbal commands with gestures to bend his fingers from the thumb in order and open his fingers bilaterally at the same time, a whole series of movements at once, he bent and extended his fingers, one at a time, alternating bilaterally with a chant to keep time (Figure 2 and Supplementary Video 6). He was aware that he could not move both sides simultaneously and substituted in-phase movements with anti-phase or unilateral movements. The patient often chanted aloud to keep time. Bimanual in-phase closing-opening movement performance improved when cued by the sounds of a metronome at 40 beats per minute (Supplementary Videos 7, 8).

As for the other parts of the body, bilateral leg movements showed similar tendency; anti-phase movements were more skillful than in-phase movements (Supplementary Videos 9, 10). There was no language impairment.

The next day (the 6th postoperative day), his bimanual movements improved compared to those on the day before; however, every movement was performed by calling out. At the beginning of the bilateral in-phase movement, the left hand was delayed in facing the palm upward and making a fist, but afterward, the opening/closing movements were smooth (Supplementary Video 11). As for the anti-phase movements, initial closing of the right hand and opening of the left hand were performed as instructed; however, the patient was unable to perform bilateral opposite movements simultaneously and made fists with both hands. Instead of pronation/supination of both forearms, the patient showed alternating unilateral movement. When instructed to perform sequential movements of the bilateral fingers, he could simultaneously flex the bilateral thumbs and index fingers; however, flexion/extension of the other fingers was performed alternately (Supplementary Video 12). Unilateral left-side movements were skillful but the actual movement performed was opposite to his call-out; e.g., he opened his palm while saying “fist.”

On the 15th postoperative day, in-phase bimanual movements, namely, closing/opening and sequential finger movements improved (Supplementary Video 13).

## 4. Discussion

**FIGURE 3:**
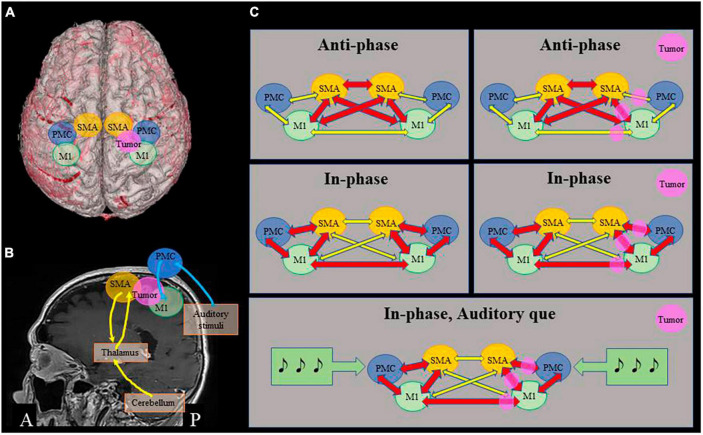
Proposed mechanisms of bimanual movement coordination. **(A)** 3D image of the patient, showing positional relationship among the tumor and motor areas. **(B)** Motor loops. Cerebellum – thalamus/basal ganglia – ventral areas of supplementary motor cortex (SMA) (yellow), and dorsal premotor cortex (PMC) – primary motor cortex (M1) associated with auditory stimuli (blue). **(C)** Schematic representations of relationship among motor cortices. In anti-phase movement, close connection among bilateral SMA and M1 is important, which is relatively preserved in this patient (upper row). In in-phase movement, connections between bilateral M1 and between SMA and PMC of each side are important, that are impaired in this patient (middle row). Auditory que stimulates bilateral dorsal PMC and ameliorates in-phase movements (lower row).

To the best of our knowledge, this is the first report to demonstrate disturbance of bimanual in-phase movement after frontal lobe surgery. While functional localization of the SMA and PMC is heterogenous among individuals (Fried et al., 1991; Chung et al., 2005; Genon et al., 2018), the current observation demonstrated that the resected area included the SMA and PMC in the superior frontal gyrus. In addition, the resection interrupted network among bilateral SMA, PMC, and primary motor cortices (Figure 3). We found a novel symptom of SMA syndrome, which also indicates an importance of disconnection of motor areas on pathophysiology of SMA syndrome.

In general, anti-phase movements are more complex and require greater activation of the SMA and PMC than in-phase movements (Sadato et al., 1997). There is a phenomenon called phase transition, which is usually observed as a change from anti-phase to in-phase transition during bimanual coordination tasks (Repp, 2005). However, in our patient, in-phase sequential bilateral finger movement was more disturbed than anti-phase movement. Transcranial magnetic stimulation of the primary motor cortex disrupts bimanual in-phase tasks, whereas bimanual anti-phase tasks remain unaffected (Chen et al., 2005). Furthermore, the interhemispheric connectivity between the primary hand motor regions as per functional magnetic resonance imaging decreases during uncoupled bilateral finger movements compared to that during synchronous movements (Meister et al., 2010). Thus, transient dysfunction and disconnection of the bilateral primary motor cortices, in addition to lesions in the SMA and PMC, likely contributed to the current findings.

In humans, the SMA plays a significant role in self-paced, signal-triggered, and sequential finger movements (Mushiake et al., 1991; Tanji, 1996; Tanji and Mushiake, 1996). Lesions in the SMA impede the selection of appropriate movements; however, external sensory cues can ameliorate task impairments in monkeys (Mushiake et al., 1991; Tanji, 1996; Tanji and Mushiake, 1996). The lateral PMC is activated by both externally triggered and self-initiated tasks (Grefkes et al., 2008; Potgieser et al., 2014). Thus, auditory cues and vocalizations helped initiate and execute bimanual movements in our patient probably via residual function of the PMC. External triggers can elicit rapid movements (kinesia paradoxica) (Jankovic, 2008) and have been utilized in rehabilitation mainly of gait disturbance in patients with Parkinson’s disease (Arias and Cudeiro, 2008). The effect of auditory cueing on bimanual coordination may be useful for the diagnosis of anatomical localization of the superior frontal gyrus and functional localization of the SMA and PMC of the lesion as well as the resected areas and for rehabilitation of patients with frontal lobe lesions or dysfunction.

Auditory cues were delivered at a tempo of 40 beats per minutes, i.e., interstimulus interval of 1.5 s. The pace is based upon the data that, when interstimulus interval range approximately from 0.6 to 1.8 s, subjects can tap their fingers synchronous to auditory stimuli integrating the timing and movements (Mates et al., 1994; Repp, 2005). There are two distinct motor loops, one is the cerebellum, thalamus/basal ganglia and ventral areas of motor cortex (primary motor cortex, SMA and ventral PMC), and the other is dorsal PMC and primary motor cortex (Middleton and Strick, 2000; Figure 3). It is hypothesized that phase correction of bimanual movement according to auditory stimuli in relatively slow pace is associated with the latter loop via dorsal PMC, whereas pace correction, especially rapid movement execution, is associated with ventral motor loop (Repp, 2005).

Impairment of bimanual movement in our patient fulfils operational definitions of apraxia; failure to produce the correct movement in response to a verbal command, and failure to imitate correctly a movement performed by the examiner (Leiguarda and Marsden, 2000). The characteristics of fine finger movement deficits can be categorized as a specific form of limb-kinetic apraxia with preserved muscle strength or perception of various senses. Importance of early diagnosis and treatment intervention of limb-kinetic apraxia using rehabilitation is recently drawing attention in poststroke patients (Jang and Byun, 2022).

## Patient perspective

The present observations demonstrate that in addition to the disturbance of anti-phase bimanual movements, resection of the frontal lobe involving the SMA can cause transient impairment of in-phase bimanual diadochokinesis, which can be more severe than the impairment of anti-phase movements. The effect of auditory cueing on bimanual skills may be useful in the diagnosis of anatomical and functional localization of the lesion and in rehabilitation of patients with brain tumors, as in the case of degenerative movement disorders.

## Data availability statement

The raw data supporting the conclusions of this article will be made available by the authors, without undue reservation.

## Ethics statement

The requirement of ethical approval was waived by the Ethics Committee, Nagahama City Hospital. The studies were conducted in accordance with the local legislation and institutional requirements. Written informed consent was obtained from the individual(s) for the publication of any potentially identifiable images or data included in this article.

## Author contributions

KT: conceptualization, investigation, resources, data curation, and writing – original draft. TY and YH: conceptualization, investigation, resources, and data curation. YM: data curation and visualization. SH: investigation, resources, and data curation. MK: conceptualization, writing – review and editing, visualization, supervision, and funding acquisition. TS: investigation, resources, data curation, and supervision. All authors contributed to the article and approved the submitted version.

## Supplementary material

Supplementary Video 1 The 5th postoperative day. Instruction of unilateral left arm elevation was given with gesture, and the movement was smooth.

Supplementary Video 2 The 5th postoperative day. When he was instructed to raise his both arms (‘banzai’ in Japanese) with gesture, he raised his right arm only.

Supplementary Video 3 The 5th postoperative day. Unilateral left forearm pronation/spination was smooth.

Supplementary Video 4 The 5th postoperative day. Despite instruction of bilateral in-phase forearms pronation/spination but left movement delayed.

Supplementary Video 5 The 5th postoperative day. Sequential movements of unilateral left fingers were smooth.

Supplementary Video 6 The 5th postoperative day. When an instruction on in-phase sequential finger movement was given by verbal commands with gestures to bend his fingers from the thumb in order and open his fingers bilaterally at the same time, a whole series of movements at once, he bent and extended his fingers, one at a time, alternating bilaterally with a chant to keep time. See Figure 2.

Supplementary Video 7 The 5th postoperative day. Bilateral in-phase closing/opening (fist/palm, ‘gu/pa’ in Japanese) in his own pace was instructed. Left hand delayed at 5th turn and he performed anti-phase movement instead of in-phase movement.

Supplementary Video 8 The 5th postoperative day. The same movement as Supplementary Video 7 with metronome sound que at 40 beats per minute. Opening of 4th turn delayed but he could catch up with the original rhythm.

Supplementary Video 9 The 5th postoperative day. Anti-phase bilateral leg movement was smooth.

Supplementary Video 10 The 5th postoperative day. He was instructed to raise bilateral legs at the same time, but raised his right leg only.

Supplementary Video 11 The 6th postoperative day. Bilateral in-phase closing/opening (fist/palm) in his own pace was instructed. At the beginning of the bilateral in-phase movement, the left hand was delayed in facing the palm upward and making a fist, but afterward, the opening/closing movements were smooth. Every movement was performed by calling out.

Supplementary Video 12 The 6th postoperative day. When instructed to perform sequential movements of the bilateral fingers, he could simultaneously flex the bilateral thumbs and index fingers; however, flexion/extension of the other fingers was performed alternately.

Supplementary Video 13 The 15th postoperative day. In-phase bimanual sequential movements improved.
