# Supplementary figures and images for: Impairment of bimanual in-phase movement during recovery from frontal lobe tumor surgery: a case report
Source: Front Neurosci. 2023 Sep 28;17:1217430. doi: 10.3389/fnins.2023.1217430 (PMC10568456; doi:10.3389/fnins.2023.1217430)

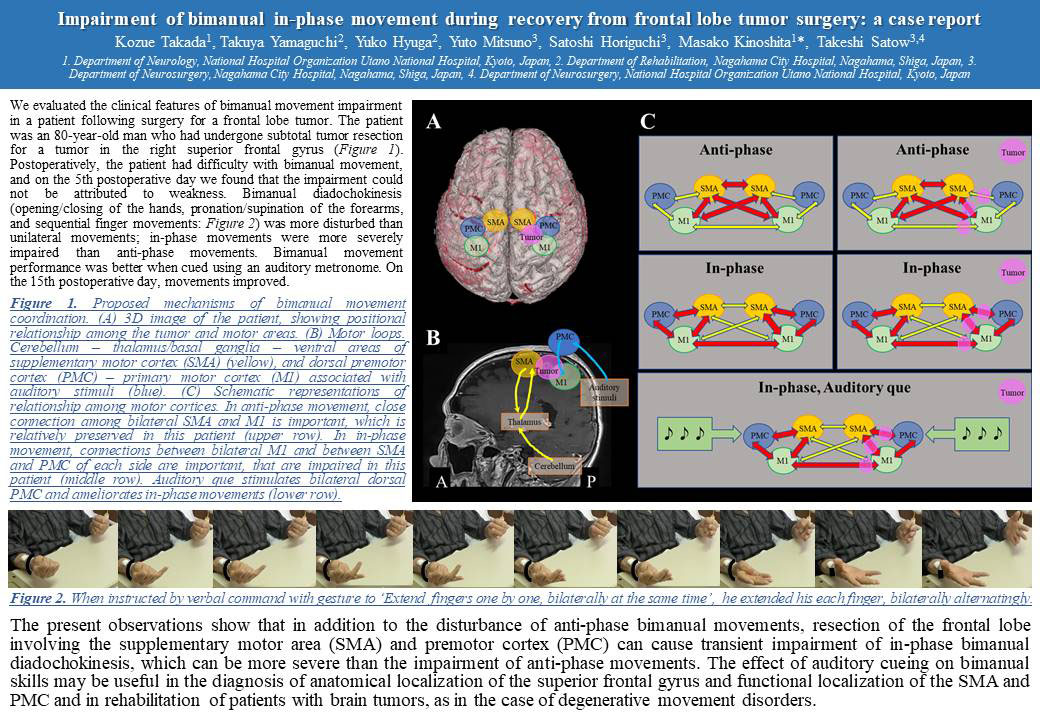

Supplement: Supplementary file 1 [file Image_1.JPEG]
